# Comparing the outcome between multicentric/multifocal breast cancer and unifocal breast cancer: A systematic review and meta-analysis

**DOI:** 10.3389/fonc.2022.1042789

**Published:** 2022-12-16

**Authors:** Yalan Zhang, Fan Liu, Qianqian Gao, Yahui Chai, Yan Ren, Hongyou Tian, Bin Ma, Ailin Song

**Affiliations:** ^1^ Department of General Surgery, Lanzhou University Second Hospital, Lanzhou University, Lanzhou, China; ^2^ Second Clinical Medical College, Lanzhou University, Lanzhou, China; ^3^ Evidence-Based Medicine Center, School of Basic Medical Sciences, Lanzhou University, Lanzhou, China; ^4^ Key Laboratory of Evidence-Based Medicine and Knowledge Translation of Gansu Province, Lanzhou University, Lanzhou, China

**Keywords:** Multicentric/multifocal breast cancer (MMBC), unifocal breast cancer (UFBC), prognosis, systematic review, meta-analysis

## Abstract

**Objective:**

This systematic review and meta-analysis compares the outcome between MMBC and unifocal breast cancer (UFBC), in order to provide a theoretical basis for the design of an appropriate clinical therapeutic strategy of MMBC patients.

**Methods:**

PubMed, Embase, The Cochrane Library, Web of science, CNKI, WanFang Data, CBM and VIP database were searched from inception to July 2021, and observational studies reporting the outcome of patients with MMBC and UFBC were included. We extracted or calculated the mortality rates of MMBC and UFBC patients; and obtained the hazard ratios; odds ratios; relative risks; and the corresponding 95% confidence intervals from the eligible studies. All the meta-analyses were conducted by using the Stata 15.0 software.

**Results:**

31 eligible studies comprising a total of 15,703 individuals were included. The meta-analysis revealed that MMBC did not have a significant association with poor overall survival (*HR*=1.04, 95% *CI*=0.96-1.12), disease-free survival (*HR*= 1.07, 95% *CI*= 0.84-1.36), breast cancer-specific survival (*HR*=1.42, 95% *CI*= 0.89-2.27), recurrence-free survival (*HR*= 0.878, 95% *CI*= 0.652-1.182), local recurrence-free survival (*HR*= 0.90, 95% *CI*= 0.57-1.42), and contralateral breast cancer risk (*RR*= 0.908, 95% *CI*= 0.667-1.234). However, MMBC appeared to have a correlation with a slightly higher risk of death (*OR*=1.31, 95% *CI*=1.18-1.45).

**Conclusion:**

Patients with MMBC appeared to have a higher risk of death, however, it may not be independently associated with poorer outcomes. Considering the inter-study heterogeneity and other limitations, our results need to be validated by further multicenter prospective studies with a large sample size in the future.

## 1 Introduction

In 2020, the estimated number of new breast cancer cases was about 2.26 million and cancer deaths were projected to be around 0.68 million worldwide (1). Globally, breast cancer is one of the most common cancers and the most frequent cause of cancer death among women.

Breast cancer usually presents as a single lesion, but in unilateral breast cancer, multiple lesions may appear simultaneously or sequentially. To enable further study and differentiate it from the subtypes with only one separate lesion - unifocal breast cancer (UFBC), researchers have subdivided such cases into two categories. The first one is multicentric breast cancer (MCBC), wherein two or more tumors are present in more than one quadrant of the same breast, but some researchers suggest that regardless of whether the different lesions are present in multiple quadrants of the same breast, those separated by >4-5 cm from each other should be called MCBC (2, 3). The second one is multifocal breast cancer (MFBC), wherein two or more tumors are found in the same quadrant of the breast (4, 5). Regarding the minimum distance between the MFBC lesions, Lüttges et al. (6) suggested that it should be at least 2 cm, while Ustaalioglu et al. (7) suggested that the spacing distance over 1 mm was enough. However, other investigators suggested that independent lesions in the specimen needed to be observed by the naked eye (disregarding microscopic lesions) (3, 8).Moreover, others indicated that multiple lesions should be clearly separated by non-cancerous tissue or carcinoma *in situ* (5, 8–11). Considering the difficulties with measurement and precision, these two categories are often studied together, and called multicentric/multifocal breast cancer (MMBC) (9).

At present, the prevalence of MMBC ranges between 6% - 77% (5, 12–14). Although MMBC is a common occurrence, its clinicopathological characteristics, precise therapeutic strategies, and prognosis and survival are not well characterized. Past studies have shown that MMBC was correlated with an increase in the lymph-node involvement, less differentiation, *HER*-2 positivity and lymphovascular invasion as compared to UFBC (4, 13, 15). In terms of the prognosis, many studies have explored the differences between MMBC and UFBC, but the findings have been largely inconclusive. Some studies have shown that MMBC patients had a higher mortality rate and shorter survival than the UFBC patients, and suggested that MMBC as an independent prognostic risk factor (4, 16, 17). However, others reported that MMBC patients had a similar prognosis as the UFBC patients (5, 10, 18), in terms of the OS and the DFS. The eighth edition of the AJCC TNM staging system of breast cancer indicates that the overall prognostic impact of smaller lesions on MMBC is not considered. However, the guidelines also emphasize the importance of a comprehensive judgment in the real clinical practice, especially when synchronous invasive tumors are identified (19). Therefore, there are conflicting reports regarding the prognosis of MMBC and UFBC patients, and whether MMBC is associated with a poorer prognosis is controversial (4, 5, 9, 16, 17, 20–24).

Thus, the current study summarizes the studies related to the comparison of prognosis between MMBC and UFBC patients, and synthesizes a systematic review and meta-analysis to evaluate the differences in the prognosis, in order to provide a theoretical basis for the design of an appropriate therapeutic strategy for treating MMBC patients.

## 2 Methods

The study was conducted in accordance with the Preferred Reporting Items for Systematic Reviews and Meta-Analysis (PRISMA) guidelines (25).

### 2.1 Eligibility criteria

The inclusion criteria for the study were as follows: (1) Participants: female patients with pathologically proven stages I-III of unilateral invasive breast cancer, aged ≥18 years, without contralateral breast cancer, without distant metastases, without any previous or concomitant malignant disease, without any limitation due to race or nationality; (2) Exposure: patients with clinically or image-based or pathologically diagnosed MMBC or UFBC; (3) Outcomes: mortality rates of MMBC and UFBC, overall survival (OS), disease-free survival (DFS), breast cancer-specific survival (BCSS), recurrence-free survival (RFS), local recurrence-free survival (LRFS), risk of contralateral breast cancer (CBC); (5) Type of study: case-control and cohort studies.

The exclusion criteria were as follows: (1) articles without a clear definition of MMBC; (2) MFBC or MCBC only; (3) duplicate articles; (4) articles published in languages other than English or Chinese.

### 2.2 Information sources and search strategy

The following eight electronic databases were independently searched by two researchers (YLZ and FL) and the timeline was set at July 2021: PubMed; Embase; the Cochrane Library; Web of Science; CNKI; WanFang Data; CBM; and the VIP database. The references of the included studies and previous MMBC related systematic reviews were also checked, and the relevant literature was manually added if available. Before the final analyses, we re-searched the literature to ensure that any study meeting the inclusion criteria was included as far as possible. The detailed search strategies are showed in **Appendix Table 1**.

### 2.3 Study selection

Two researchers (YLZ and FL) checked all the collected studies independently and if there was any disagreement between them, a discussion or the third reviewer (QQG)’s decision was taken into account. All the retrieved literature were imported into Endnote X9 software. After removing the duplicates, we firstly screened the articles by the title and abstract and then identified the final included studies through the full-text reading of previously screened literature. Then, we recorded the reasons for excluding the literature in the last two steps.

### 2.4 Data collection

Three researchers were involved in the data collection task. Two of them (YLZ and FL) independently collected the data from the included studies and recorded them in a pre-defined spreadsheet by using the Microsoft Excel 2021 software. The differences of opinion were discussed, and if they were still unresolved, a third Reviewer (QQG)’s opinion was taken into account. We extracted the following information from the included studies: (1) the first author’s name and the publication year, region where the study was conducted, study design, and recruitment period; (2) the sample size and age; (3) follow-up time; (4) definition of MMBC; (5) the AJCC edition used for the T-staging; (7) mortality rates of MMBC and UFBC patients. If the data needed further confirmation, the corresponding author of the article was contacted by email.

### 2.5 Quality assessment

The risk of bias was assessed by using the Newcastle-Ottawa Scale (NOS) (26), whose full mark was 9, a score of 8 to 9 was considered as low risk of bias (high quality literature), a score of 5~7 was considered as moderate risk (moderate quality literature), and a score of 0~4 was considered as high risk (low quality literature). The risk of bias was independently assessed by two researchers (YLZ and FL) and the discrepancies were resolved by discussion or a third reviewer’s (QQG) decision was taken into account.

### 2.6 Statistical analysis

In this study, we extracted or calculated the mortality rates for MMBC and UFBC patients, and the *HRs*, *RRs* and *ORs* with the corresponding 95% *CIs* were obtained from the multivariate analyses of the included studies. If two or more studies reported the data of an outcome, a meta-analysis was performed, otherwise, only a descriptive analysis was performed. All the meta-analyses were completed by using the Stata 15.0 software. The Cochrane’s Q-test was applied to evaluate the inter-study heterogeneity and the *I^2^* statistic was used to quantify the degree of heterogeneity. If the studies were without statistical heterogeneity, the meta-analysis was conducted using the fixed-effects model. If *I^2^* ≥50% and *P*<0.10, it indicated that there was a significant and substantial heterogeneity (27) among the studies, and hence a random-effects model was employed after excluding the significant clinical heterogeneity. When there was a significant clinical heterogeneity, sensitivity and subgroup analyses were used, or only a descriptive analysis was performed. *P*<0.05 was considered as statistically significant. The publication bias was evaluated using funnel plots or the Egger’s test when over 10 studies were included. When *P* > 0.05, it suggested the absence of publication bias. And if there was a publication bias, the trim-and-fill method was used to assess the further effect of publication bias on the results.

## 3 Results

### 3.1 Search results

**Figure 1:**
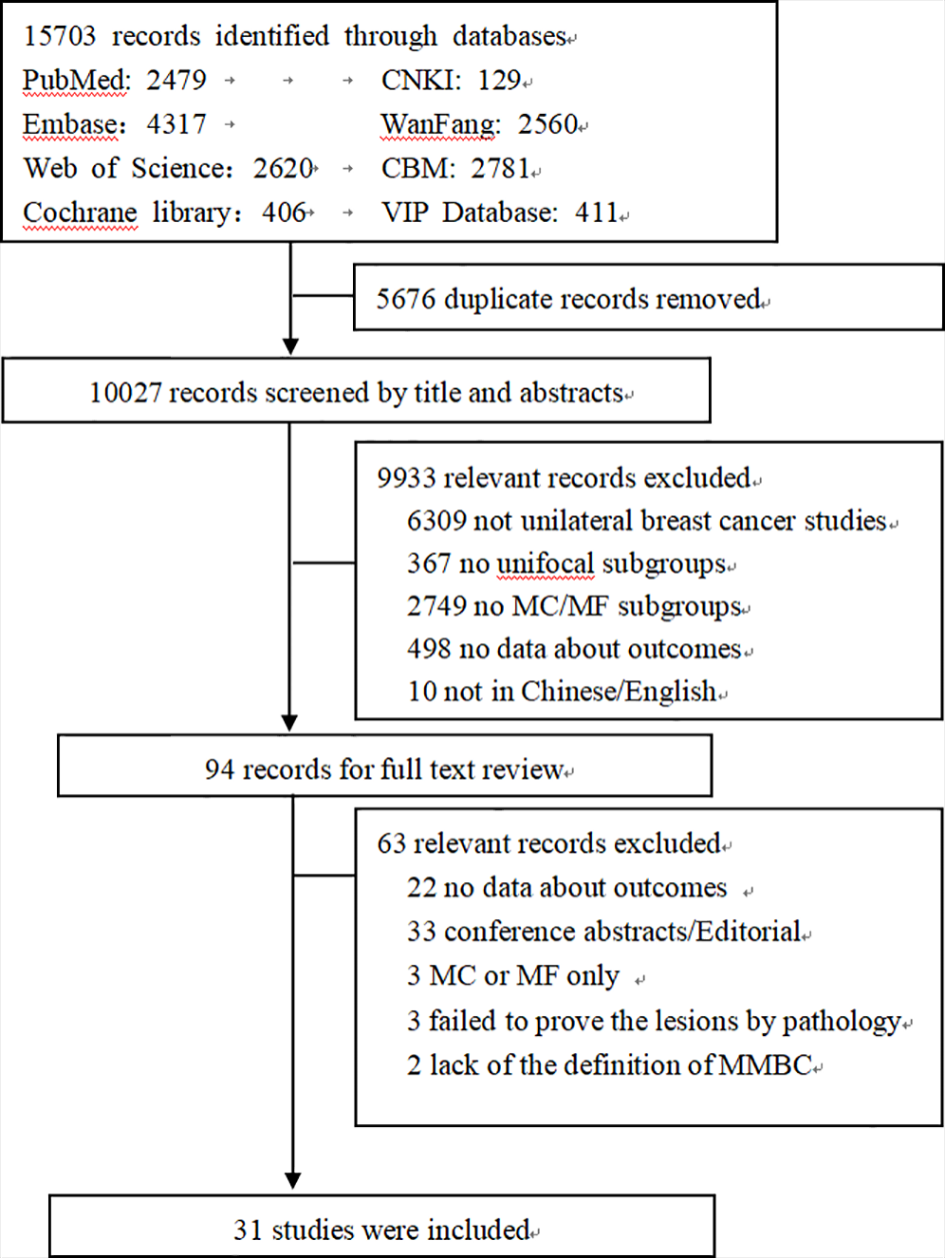
Flowchart of literature selection.

The eight databases were searched and a total of 15,703 articles were retrieved. After removing the duplicate articles, 10,027 were available for further screening. 9933 articles were excluded after browsing the titles and abstracts, and the full-text was examined for 94 studies. Lastly, 31 articles (2–5, 7–11, 13, 14, 16–18, 20–24, 28–39) met the eligibility criteria mentioned previously. The detailed selection procedures and statistics are shown in **Figure 1**.

### 3.2 Study characteristics

Among the included studies, four (5, 14, 18, 39) were prospective cohort studies, twenty-seven (2–4, 7–11, 13, 16, 17, 20–24, 28–38) were retrospective cohort studies, and one (23) was a retrospective age-matched cohort study. The total number of participants was 88,147 and the sample sizes ranged from 118 (29) to 25,320 (14). The follow-up for fifteen studies was over 60 months (4, 8–11, 14, 17, 20, 23, 29, 31, 33, 34, 36, 39), and seven studies were published within 5 years (5, 9, 21–23, 33, 35). The detailed information is presented in **Appendix Table 2**.

### 3.3 Assessment of the quality of the included articles

The quality of the included studies was evaluated using the NOS scale. Seventeen studies (3, 4, 7, 9–11, 14, 16, 17, 23, 28, 29, 33–35, 38, 39) were of high quality, while fourteen (2, 5, 8, 13, 18, 20–22, 24, 30–32, 36, 37) were of moderate quality and none of the study was of low quality. The details are listed in **Appendix Figure 1**.

### 3.4 Outcomes

#### 3.4.1 Overall survival

**Figure 2:**
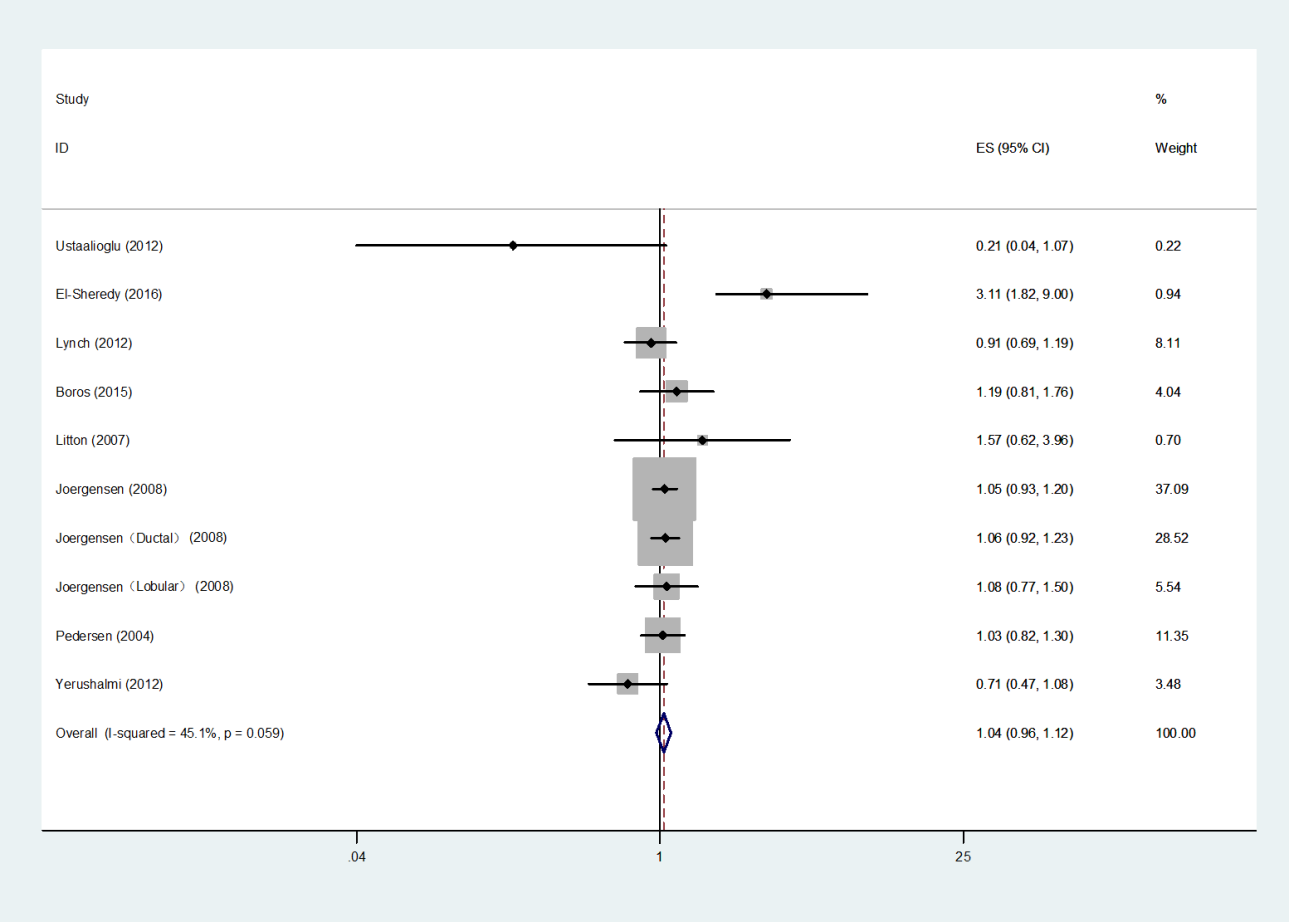
Forest plot of OS comparing MMBC and UFBC.

9 studies (7, 10, 13, 16, 20, 21, 30, 32, 39) were enrolled in the analysis of OS, 8 (7, 10, 13, 16, 20, 30, 32, 39) of them reported *HRs* and 1 (21) reported *OR*. The heterogeneity test was not statistically significant (*I^2 =^* 45.1%, *P*=0.059), and 8 *HRs* were selected for the meta-analysis using the fixed-effects model. The analysis revealed no statistically significant relationship between MMBC and poor OS in the multivariate analysis (*HR*=1.04, 95% *CI*= 0.96-1.12, *I^2 =^* 45.1%, *P*=0.059, 8 studies) (**Figure 2**). Moreover, Djordjevic-Jovanovic et al. (21) reported no remarkable difference in the 5-year OS between UFBC and MMBC patients in a multivariate analysis (*OR*=0.91, 95% *CI*=0.65-1.21, *P*=0.51).

#### 3.4.2 Disease-free survival

**Figure 3:**
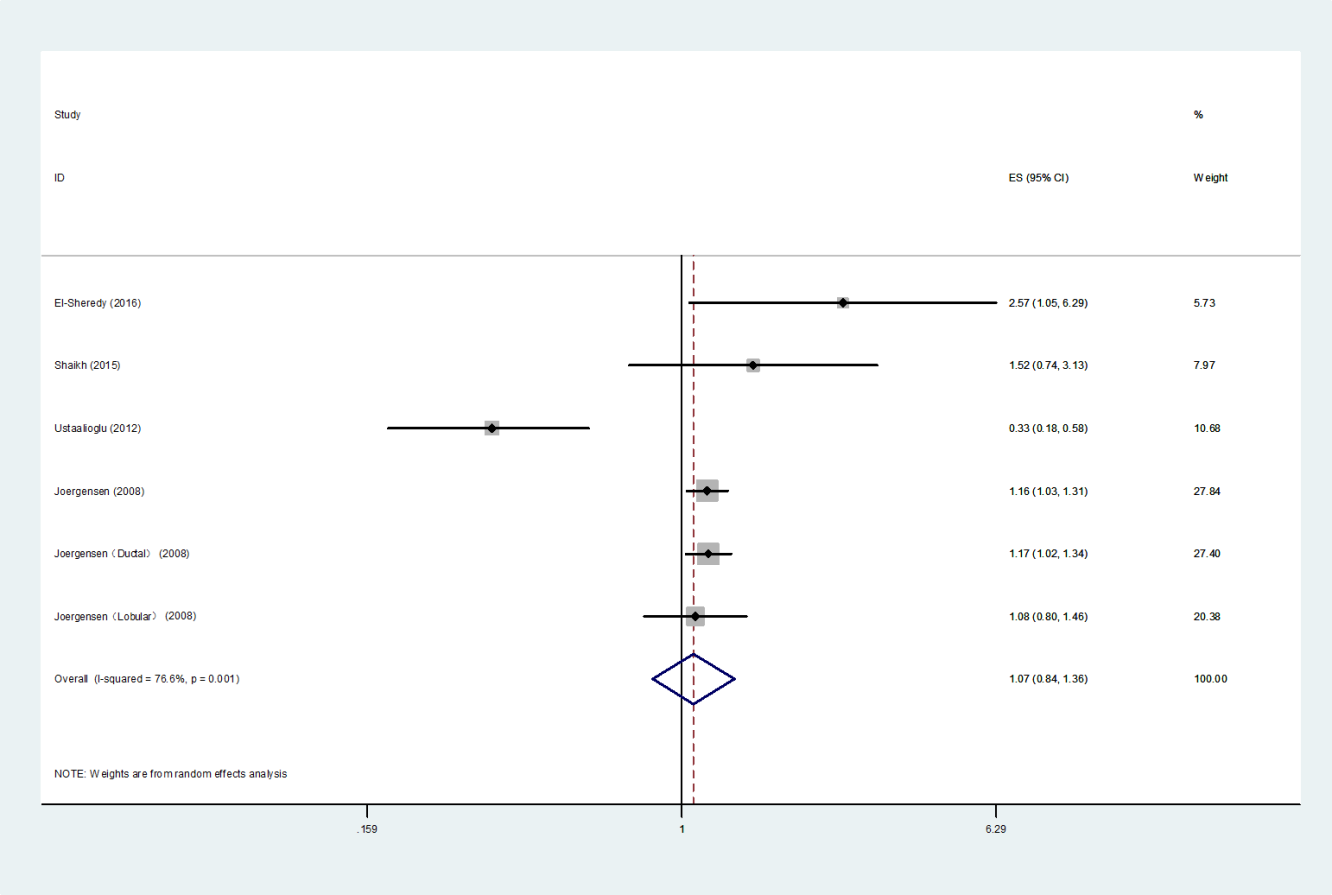
Forest plot of DFS comparing MMBC and UFBC.

In total, four studies (7, 16, 24, 30) were integrated into the *HR*s analysis of DFS. Heterogeneity tests showed statistical significance and therefore a random effects model was applied. The results indicated that compared to UFBC, MMBC was not associated with poorer DFS by multivariate analysis (*HR*=1.07, 95% *CI*=0.84-1.36, *I^2 =^* 76.6%, *P*=0.001, 4 studies) (**Figure 3**).

#### 3.4.3 Breast cancer specific survival

**Figure 4:**
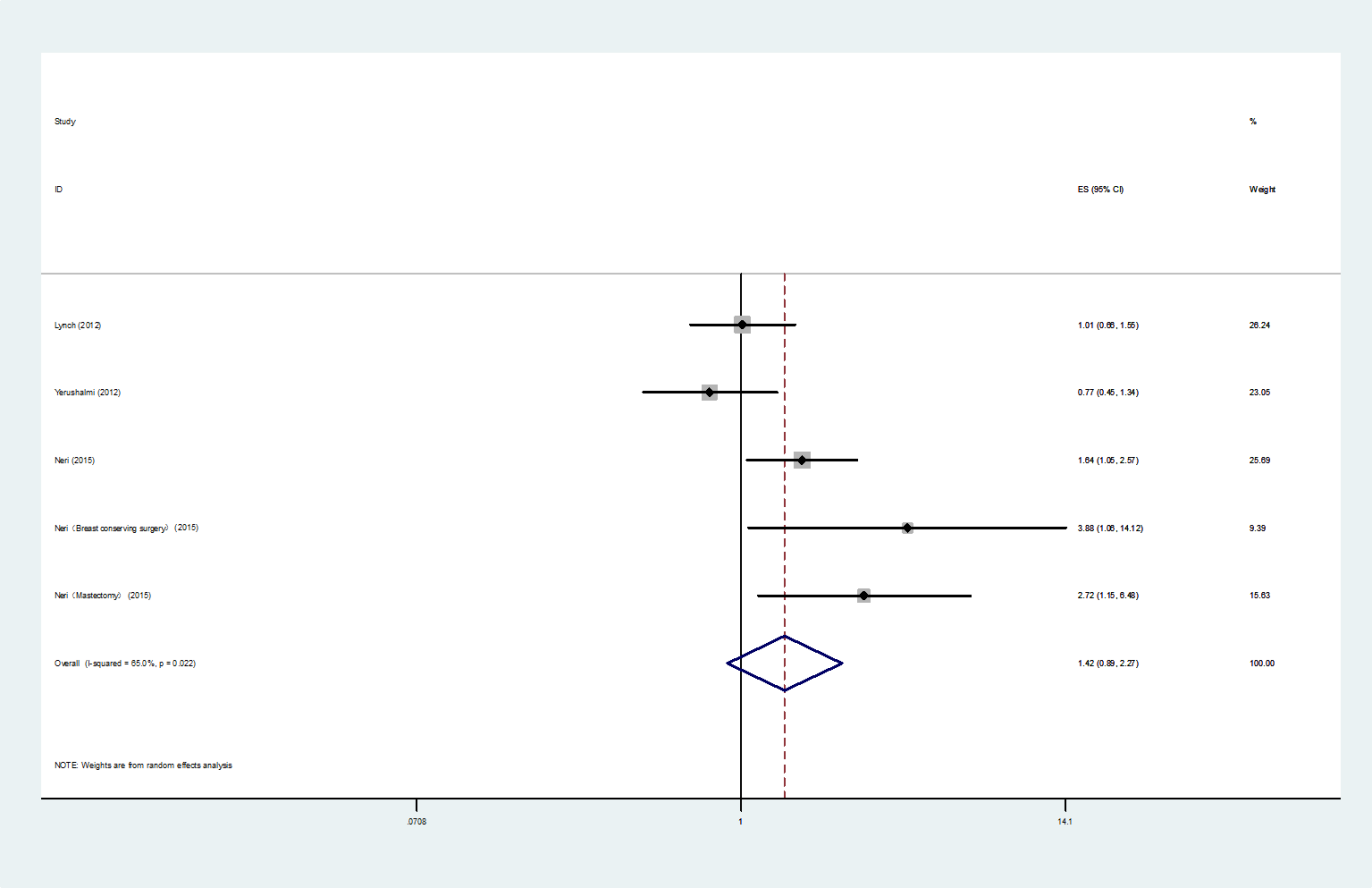
Forest plot of BCSS comparing MMBC and UFBC.

Four studies (4, 13, 17, 39) were included for the analysis of BCSS, 3 studies (13, 17, 39) reported *HRs*, and 1 study (4) reported *RR*. The 3 *HRs* were selected for the meta-analysis, and the heterogeneity was noticeable (*I^2 =^* 65.0%, *P*=0.022), thus we chose a random-effects model to perform the analysis. In the multivariate analysis, the meta-analysis showed that in comparison with UFBC, MMBC had no clear correlation with poorer BCSS (*HR*=1.42, 95% *CI*=0.89-2.27, 3 studies) (**Figure 4**). Moreover, Boyages et al. (4) reported four *RR* values for BCSS from multivariate analysis. They used aggregate tumor size of each foci in MMBC or the dominant tumor size of MMBC to determine the “T-stage” and set a 2 cm tumor diameter boundary. The results showed that when the tumor diameter was less than 2 cm, there was no statistical difference in the 10-year BCSS between the UFBC and MMBC patients (Dominant: *RR* (95% *CI*) =0.86 (0.39-1.87), *P*=0.695; Aggregate: *RR* (95% *CI*) =1.00 (0.36-2.76), *P*=1.00). When the tumor diameter was greater than 2 cm, the results of the aggregate tumor size staging method also indicated no significant difference between the two groups, but the largest or the dominant tumor size staging system showed a different result (Dominant: *RR* (95% *CI*) =1.91(1.15-3.16), *P*=0.012; Aggregate: *RR* (95% *CI*) =1.13(0.82-2.09), *P*=0.267).

#### 3.4.4 Recurrence-free survival

**Figure 5:**
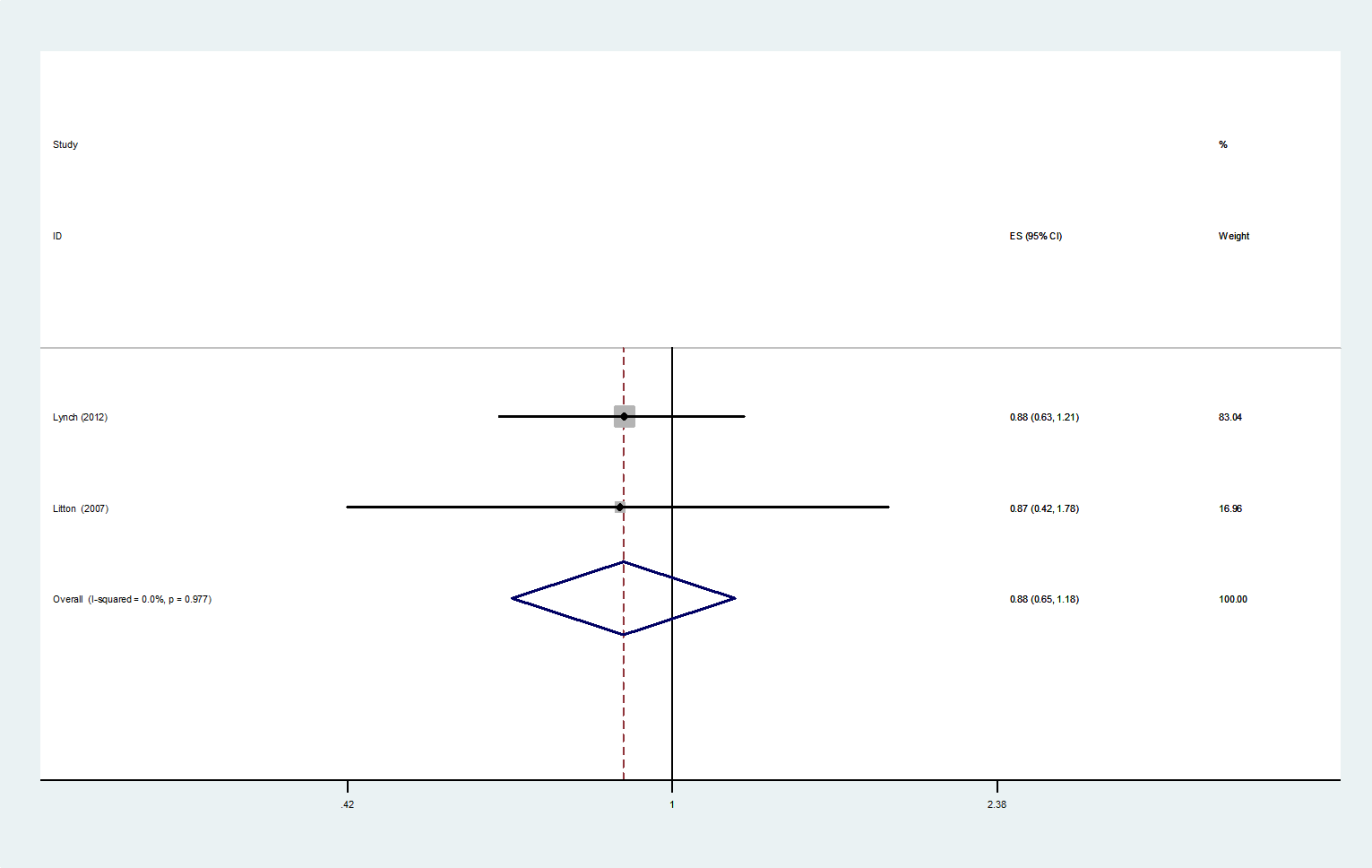
Forest plot of RFS comparing MMBC and UFBC.

The analysis of *HRs* for the RFS was comprised of two studies (13, 32). A fixed-effect model meta-analysis demonstrated that in the multivariate analysis, compared with UFBC, MMBC was not significantly associated with poorer RFS (*HR*= 0.878, 95% *CI*=0.652-1.182, *I^2 =^* 0.00%, *P*=0.977, 2 studies) (**Figure 5**).

#### 3.4.5 Local recurrence-free survival

**Figure 6:**
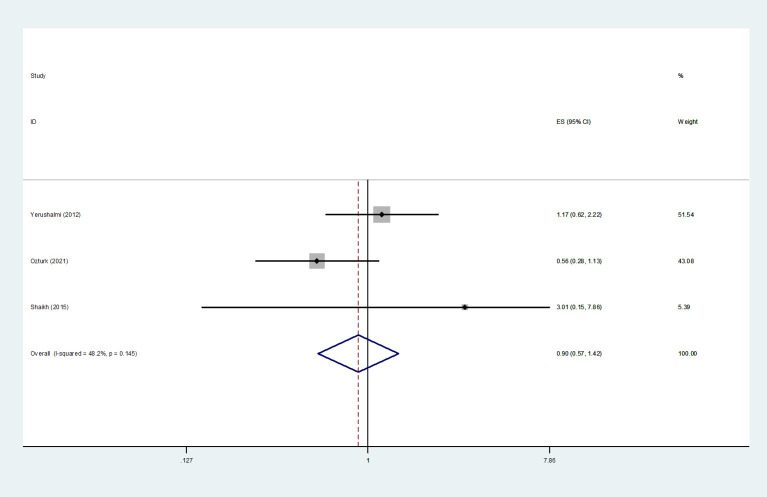
Forest plot of LRFS comparing MMBC and UFBC.

For LRFS, three studies (5, 24, 39) were included in the *HRs* meta-analysis. The results were analyzed using a fixed-effects model, and suggested that MMBC was not significantly associated with poorer LRFS by multivariate analysis (*HR*=0.90, 95% *CI*=0.57-1.42, *I^2 =^* 48.2%, *P*=0.145, 3 studies) (**Figure 6**).

#### 3.4.6 Mortality rates

**Figure 7:**
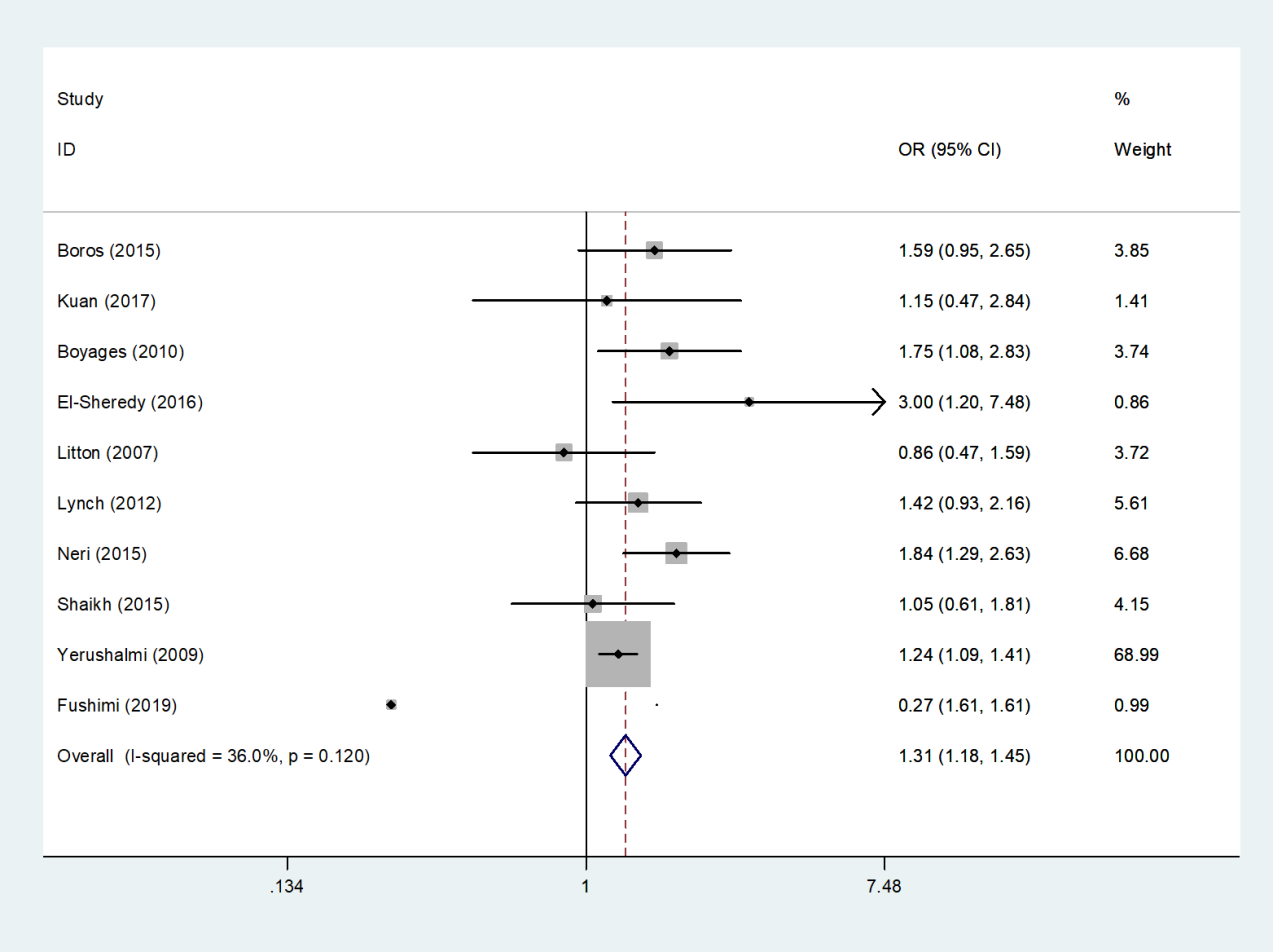
Forest plot of the mortality rates of MMBC versus UFBC patients.

10 studies (4, 9, 13, 14, 16, 17, 20, 23, 24, 32) described the mortality rates of MMBC and UFBC patients. Meta-analysis by fixed-effects model showed that in comparison with UFBC, MMBC was associated with a higher mortality (*OR*=1.31, 95% *CI*=1.18-1.45, *I^2 =^* 36.0%, *P*=0.12, 10 studies) (**Figure 7**).

#### 3.4.7 Contralateral breast cancer

Only one study (14) among the included literature reported the multivariate analysis results of CBC risk. Yerushalmi et al. (14) concluded that MMBC was not significantly associated with higher risk of CBC (*RR*=0.908, 95% *CI*=0.667-1.234, *P* = 0.537).

## 4 Discussion

In this review, we pooled the data for MMBC and UFBC with regards to the OS, DFS, RFS, BCSS, LRFS, mortality, and CBC aspects and performed a meta-analysis. The final results showed that MMBC patients had a similar prognosis as the UFBC patients, except for a slightly higher mortality rate.

### 4.1 MMBC patients may have a slightly higher mortality rate

In this paper, we found that MMBC patients had a slightly higher mortality rate than the UFBC patients (*OR*=1.31, 95% *CI*=1.18-1.45), which could mainly be because MMBC patients had a relatively high total tumor load and more aggressive biological behavior. In a previously published review, compared to UFBC, MMBC was shown to have a higher proportion of poorly differentiated tumors and a greater risk of vascular invasion (2), which suggested that MMBC was associated with extensive intra-ductal lesions and an invasive lobular carcinoma component, which might increase the risk of positive surgical margins (40–42). At the same time, MMBC patients were more likely to develop tumor recurrence and metastases. And Neri et al. (17) found that MMBC was associated with the absence of ER and Her2-neu positive status which may reduce the possibility of MMBC patients benefiting from endocrine therapy and targeted therapy. Moreover, Lang et al. (43) reported a higher rate of axillary lymph node metastasis and a higher Ki67 proliferation index in MMBC patients compared to the UFBC patients, which to some extent suggested that MMBC patients might have a poorer outcome. However, previous studies (23, 44) reported that when controlling for the age, there was no significant difference in mortality between UFBC and MMBC patients (5.3% versus 7%, *P* = 0.89 with a median follow-up period of 3 years and 13% versus 14.7%, *P* = 0.89 with a median follow-up period of 7 years). And it is worth noting that more MMBC patients in this cohort opted for total mastectomy, which could have provided a survival benefit for patients these patients. Additionally, Yerushalmi et al. (39) reported that patients who underwent breast-conserving surgery in stages I-II also showed a similar mortality as compared to MMBC and UFBC patients, but the MMBC group had less severe disease compared to the UFBC patients. The results for the comparison of mortality rates between the two groups in our study showed that MMBC patients had a higher risk of death, but in the early stage breast cancer, appropriate surgery and adjuvant treatment may also offer survival benefits for MMBC patients (4, 5, 39). Therefore, early screening for breast cancer is important, and timely diagnosis and early intervention may not only provide a survival benefit, but also allow some patients to be suitable for and benefit from less invasive surgical modalities.

### 4.2 MMBC per se may not represent a poorer prognosis

The findings from the current study showed that there were no significant differences between UFBC and MMBC patients in terms of the OS, DFS, RFS, BCSS, and LRFS in multivariate analysis, which may be due to the fact that MMBC per se is not associated with a worse prognosis. It was reported that the MMBC patients were younger, had larger tumors, had greater involvement of lymph nodes, and many of them were in pre-menopausal stage in comparison to the UFBC patients (11, 13, 28, 45, 46). The above risk factors made MMBC patients more likely to undergo mastectomy as well as receive more adjuvant therapy to some extent. When these factors were controlled in the multivariate analysis, most of the studies showed that MMBC no longer had an independent effect on the OS and DFS. However, some studies still found that MMBC was an independent prognostic factor for the OS and DFS in multivariate analysis (11, 16, 47). Meanwhile, the results from a previous systematic review (48) showed that MMBC was associated with poorer OS, but after excluding one study (49) with significant heterogeneity, the results no longer showed that MMBC was associated with poorer OS. Upon reviewing recent studies (5, 9, 21–23), we found that MMBC may be associated with some worse prognosis factors, but MMBC patients often had a similar prognosis as UFBC, which could be due to the advances in imaging technologies and pathological diagnostic techniques and the continuous optimization of the therapeutic options. Pre-operative breast MRI shows good utility in determining tumor boundaries and detection of additional tumor foci, and is not influenced by different histotypes, which helps to provide the best local treatment for MMBC patients (50). In the past, the majority of MMBC patients underwent mastectomy for the discerned higher risk for in-breast recurrence and less good cosmetic outcome. But in recent publications, breast conserving surgery can be performed in selected MMBC patients (51) and the use of daVinci Robot can improve cosmetic results (52).

For the multivariate analysis of RFS, LRFS and BCSS, the general trend supported the finding that MMBC patients had a similar prognosis to that of UFBC patients. However, only few studies were included in these outcome indicators, which may affects the results reliability. A study on outcomes in 1163 MFBC/UFBC patients reported that MFBC was independently significantly associated with LRFS, DFS, and OS, but this study did not adjust for pathologic stage, T stage and nodal status (49). Thus, further prospective studies with larger samples are needed to confirm the above findings.

Our meta-analysis results on DFS and BCSS indicated a significant and substantial heterogeneity among the included studies. And we think clinical factors cause heterogeneity mainly. With fewer studies included under each outcome indicators, the subgroup analysis may not produce meaningful results. But a decreased heterogeneity was also seen when we attempted to perform subgroup analysis based on some clinical factors (**Appendix Figure 2**).

### 4.3 MMBC may not increase the risk of CBC

Among the included studies, only one study (14) reported the results related to the development of contralateral breast cancer in multivariate analysis. And the results supported the opinion that MMBC was only a representative of intra-mammary spreading, whereas CBC was an independent event. This finding may help to alleviate anxiety and panic among the patients with MMBC, as some patients may receive excessive treatment or even make a hasty decision to undergo prophylactic surgery after the diagnosis owing to their fear of developing CBC. Moreover, the study by Kurtz et al. (31) also showed a similar probability of CBC in MMBC and UFBC (3% versus 4%) patients. However, some of the current tools to assess the risk of CBC also incorporate MMBC as a risk term and have shown a better predictive power (53). There isn’t enough evidence regarding the association between MMBC and CBC, and it is hoped that more original studies will report relevant data to support CBC-related analysis.

### 4.4 The prognostic role of the remaining lesions in MMBC needs further investigation

Breast cancer is a heterogeneous group of diseases with regard to clinical manifestation, tumor morphology, and immunohistochemical differences within tumors (54). And a recent publication emphasized that MMBC has a higher risk of metastasis, recurrence, and a worse prognosis, compared to UFBC with similar staging (TNM), and sometimes the largest one is not always the most aggressive one, and more than one tumor should be evaluated (55). Data from Boyages et al. (4) on BCSS showed that the use of different criteria for assessing the tumor T-staging could influence the final results, which showed that for tumors >20 mm in diameter, MMBC was associated with poorer BCSS after using the largest or dominant tumor size of MMBC to assess the T-staging. On the other hand, when the aggregated diameter of the lesions were used to assess the tumor staging, MMBC and UFBC patients were found to have a similar prognosis. However, Duraker et al. (2) reported that MMBC and UFBC patients had similar prognosis regardless of whether the T-staging was assessed by using the largest tumor diameter or the aggregated diameter of all the lesions. Also, several studies (13, 28) have concluded that the current TNM staging could be a good assessment of MMBC tumor load, and also showed that the difference in the overall prognosis between MMBC and UFBC patients was not statistically significant. However, it is worth noting that Fushimi et al. (9) reported that MMBC was not associated with a worse prognosis, but at the same time showed that MMBC was a major prognostic factor for DFS after assessing the T-staging using the aggregated diameter of the lesions (*HR*= 2.710, 95% *CI*= 1.011-7.264, *P*= 0.048). Therefore, the method for assessing the T-stage of MMBC may influence the results for prognosis in multivariate analyses, and the prognostic impact of the remaining lesions in MMBC requires further investigation.

### 4.5 Strengths and limitations

In this systematic review and meta-analysis, several studies were included to assess the difference in the prognosis between MMBC and UFBC patients. Here, we searched eight databases using a relatively broad terminology and our search strategy ensured as far as possible, that none of the potentially relevant studies were excluded. However, the current study suffers from some limitations. First, most of the included studies were retrospective cohort studies which may existed selection bias and data analysis bias. Second, the definition and diagnostic criteria of MMBC was not completely consistent across all the included studies. These discrepancies affected the detection of MMBC, and it could have affected the reliability of the meta-analysis results. Third, as the heterogeneity among the included studies were significant and fewer studies were included under some of the outcome indicators, the source of heterogeneity was difficult to determine and limited the accuracy of our findings further. Finally, the limitation of the choice of language could have increased the publication or language bias.

## 5 Conclusions

In summary, patients with MMBC appeared to have a higher risk of death, however, it may be not independently associated with poor OS, DFS, RFS, BCSS, LRFS, and CBC risk. With appropriate surgical interventions and adjuvant therapies, the prognosis of patients with MMBC and UFBC was similar, but the prognostic impact of every lesion in MMBC still needs further investigation. Further multicenter prospective studies with larger sample size are needed for validating the findings from the current study.

## Data availability statement

The original contributions presented in the study are included in the article/**Supplementary Material**. Further inquiries can be directed to the corresponding author.

## Author contributions

All authors contributed to the study conception and design. Study selection, data extraction and analysis were performed by YZ, FL and QG. The first draft of the manuscript was written by YZ and all authors commented on previous versions of the manuscript. All authors contributed to the article and approved the submitted version.
