# Supplementary material for: Comparing the outcome between multicentric/multifocal breast cancer and unifocal breast cancer: A systematic review and meta-analysis
Source: Front Oncol. 2022 Dec 16;12:1042789. doi: 10.3389/fonc.2022.1042789 (PMC9801517; doi:10.3389/fonc.2022.1042789)
Supplement: Supplementary file 1 [file Table_1.doc]

**Appendix Table 1 The searching strategy of each database**

| **No.** | **Databases and access** | **Search terms** |
| --- | --- | --- |
| 1 | PubMed  (via pubmed.ncbi.nlm.nih.gov) | #1 (((((((((((((((((((Breast Neoplasms[Title/Abstract]) OR (Breast cancer[Title/Abstract])) OR (Breast carcinoma[Title/Abstract])) OR (breast tumor[Title/Abstract])) OR (breast tumour[Title/Abstract])) OR (breast neoplasm[Title/Abstract])) OR (breast malignancy[Title/Abstract])) OR (breast neoplasm∗[Title/Abstract])) OR (breast tumor∗[Title/Abstract])) OR (breast carcinoma∗[Title/Abstract])) OR (breast cancer∗[Title/Abstract])) OR (breast tumour∗[Title/Abstract])) OR (mammary neoplasm∗[Title/Abstract])) OR (mammary tumor∗[Title/Abstract])) OR (mammary carcinoma∗[Title/Abstract])) OR (mammary cancer∗[Title/Abstract])) OR (mammary tumour∗[Title/Abstract])) OR (breast adenocarcinoma∗[Title/Abstract])) OR (breast carcinogenesis[Title/Abstract])) OR (breast sarcoma∗[Title/Abstract])  #2 “Breast Neoplasms”[MeSH Terms]  #3 #1 OR #2  #4 (((((((multifocality[Title/Abstract]) OR (multifocal[Title/Abstract])) OR (multicentricity[Title/Abstract])) OR (multicentric[Title/Abstract])) OR (unifocal[Title/Abstract])) OR (unifocality[Title/Abstract])) OR (unicentricity[Title/Abstract])) OR (unicentric[Title/Abstract])  #5 #3 AND #4 |
| 2 | Web of Science  (via webofscience.com) | #1 Topic:(Breast Neoplasms OR Breast cancer OR Breast carcinoma OR breast tumor OR breast tumour OR breast neoplasm OR breast malignancy OR breast neoplasm∗OR breast tumor∗OR breast carcinoma∗OR breast cancer∗OR breast tumour∗OR mammary neoplasm∗OR mammary tumor∗OR mammary carcinoma∗ OR mammary cancer∗ OR mammary tumour∗ OR breast adenocarcinoma∗ OR breast carcinogenesis OR breast sarcoma∗  #2 Topic:(multifocality OR multifocal OR multicentricity OR multicentric OR unifocal OR unifocality OR unicentricity OR unicentric)  #3 #1 AND #2 |
| 3 | EMbase  (via embase. com) | #1 'Breast Neoplasms':ti,ab,kw OR 'Breast cancer':ti,ab,kw OR 'Breast carcinoma':ti,ab,kw OR 'breast tumor':ti,ab,kw OR' breast tumour ':ti,ab,kw OR' breast neoplasm':ti,ab,kw OR' breast malignancy':ti,ab,kw OR 'breast neoplasm∗':ti,ab,kw OR 'breast tumor∗':ti,ab,kw OR' breast carcinoma∗':ti,ab,kw OR 'breast cancer∗':ti,ab,kw OR 'breast tumour∗':ti,ab,kw OR 'mammary neoplasm∗':ti,ab,kw OR 'mammary tumor∗':ti,ab,kw OR 'mammary carcinoma∗':ti,ab,kw OR 'mammary cancer∗':ti,ab,kw OR 'mammary tumour∗':ti,ab,kw OR 'breast adenocarcinoma∗':ti,ab,kw OR 'breast carcinogenesis':ti,ab,kw OR 'breast sarcoma∗':ti,ab,kw  #2 'multifocality':ti,ab,kw OR 'multifocal':ti,ab,kw OR 'multicentricity':ti,ab,kw OR 'multicentric':ti,ab,kw OR 'unifocal':ti,ab,kw OR' unifocality':ti,ab,kw OR 'unicentricity':ti,ab,kw OR 'unicentric':ti,ab,kw  #3 'breast tumor'/exp  #4 #1 OR #3  #5 #2 AND #4 |
| 4 | The Cochrane Library  (via cochranelibrary.com) | #1 (Breast Neoplasms OR Breast cancer OR Breast carcinoma OR breast tumor OR breast tumour OR breast neoplasm OR breast malignancy OR breast neoplasm∗OR breast tumor∗OR breast carcinoma∗OR breast cancer∗OR breast tumour∗OR mammary neoplasm∗OR mammary tumor∗OR mammary carcinoma∗OR mammary cancer∗OR mammary tumour∗OR breast adenocarcinoma∗OR breast carcinogenesis OR breast sarcoma∗):ti,ab,kw  #2 MeSH descriptor: [Breast Neoplasms] explode all trees  #3 (multifocality OR multifocal OR multicentricity OR multicentric OR unifocal OR unifocality OR unicentricity OR unicentric):ti,ab,kw  #4 #1 OR #2  #5 #3 AND #4 |
| 5 | CNKI  (via cnki.net) | 关键词:(乳腺癌 or 乳腺恶性肿瘤 or 乳房癌 or 乳癌 or 乳腺肿瘤 or 乳房肿瘤 or 乳房恶性肿瘤) and 关键词:(多发性 or 多灶性 or 多发 or 多病灶 or 多中心 or 多中心性 or 单发性 or 单灶性 or 单发 or 单病灶 or 单中心 or 单中心性) or 关键词:(多中心乳腺癌 or 多灶乳腺癌 or 多发乳腺癌 or 多病灶乳腺癌 or 多发性乳腺癌 or 多中心性乳腺癌 or 单中心乳腺癌 or 单灶乳腺癌 or 单发乳腺癌 or 单病灶乳腺癌 or 单发性乳腺癌 or 单中心性乳腺癌 or 同时性单侧乳腺癌) |
| 6 | CBM  (via sinomed.ac.cn) | #1 “乳腺癌” [关键词:智能] OR“乳腺恶性肿瘤”[关键词:智能] OR“乳房癌”[关键词:智能] OR“乳癌”[关键词:智能] OR“乳腺肿瘤”[关键词:智能] OR“乳房肿瘤”[关键词:智能] OR“乳房恶性肿瘤”[关键词:智能]  #2 “乳腺恶性肿瘤”[不加权:扩展]  #3 (#1) OR (#2)  #4 “多发性”[关键词:智能] OR“多灶性”[关键词:智能] OR“多发”[关键词:智能] OR“多病灶”[关键词:智能] OR“多中心”[关键词:智能] OR“多中心性”[关键词:智能] OR“单发性”[关键词:智能] OR“单灶性”[关键词:智能] OR“单发”[关键词:智能] OR“单病灶”[关键词:智能] OR“单中心”[关键词:智能] OR“单中心性”[关键词:智能]  #5 (#3) AND (#4)  #6 “多中心乳腺癌”[关键词:智能] OR“多灶乳腺癌”[关键词:智能] OR“多发乳腺癌”[关键词:智能] OR“”[关键词:智能] OR“多病灶乳腺癌”[关键词:智能] OR“多发性乳腺癌”[关键词:智能] OR“多中心性乳腺癌”[关键词:智能] OR“单中心乳腺癌”[关键词:智能] OR“单灶乳腺癌”[关键词:智能] OR “单发乳腺癌”[关键词:智能] OR“单病灶乳腺癌”[关键词:智能] OR“单发性乳腺癌”[关键词:智能] OR“单中心性乳腺癌”[关键词:智能] OR“同时性单侧乳腺癌”[关键词:智能]  #7 (#5) OR (#6) |
| 7 | WanFang Data  (via wanfangdata.com.cn) | 题名或关键词:(乳腺癌 or 乳腺恶性肿瘤 or 乳房癌 or 乳癌 or 乳腺肿瘤 or 乳房肿瘤 or 乳房恶性肿瘤) and 题名或关键词:(多发性 or 多灶性 or 多发 or 多病灶 or 多中心 or 多中心性 or 单发性 or 单灶性 or 单发 or 单病灶 or 单中心 or 单中心性) or 题名或关键词:(多中心乳腺癌 or 多灶乳腺癌 or 多发乳腺癌 or 多病灶乳腺癌 or 多发性乳腺癌 or 多中心性乳腺癌 or 单中心乳腺癌 or 单灶乳腺癌 or 单发乳腺癌 or 单病灶乳腺癌 or 单发性乳腺癌 or 单中心性乳腺癌 or 同时性单侧乳腺癌) |
| 8 | Chinese Scientific Journals Full-Text  (via cqvip.com) | 题名或关键词: (乳腺癌 or 乳腺恶性肿瘤 or 乳房癌 or 乳癌 or 乳腺肿瘤 or 乳房肿瘤 or 乳房恶性肿瘤) and 题名或关键词:(多发性 or 多灶性 or 多发 or 多病灶 or 多中心 or 多中心性 or 单发性 or 单灶性 or 单发 or 单病灶 or 单中心 or 单中心性) or 题名或关键词:(多中心乳腺癌 or 多灶乳腺癌 or 多发乳腺癌 or 多病灶乳腺癌 or 多发性乳腺癌 or 多中心性乳腺癌 or 单中心乳腺癌 or 单灶乳腺癌 or 单发乳腺癌 or 单病灶乳腺癌 or 单发性乳腺癌 or 单中心性乳腺癌 or 同时性单侧乳腺癌) |

The following terms and free words were used: breast cancer; breast carcinoma; breast tumor; breast tumour; breast neoplasm; breast neoplasms; breast malignancy; breast neoplasm*; breast tumor*; breast carcinoma*; breast cancer*; breast tumour*; mammary neoplasm*; mammary tumor*; mammary carcinoma*; mammary cancer*; mammary tumour*; breast adenocarcinoma*; breast carcinogenesis; breast sarcoma*; multifocality; multifocal; multicentricity; multicentric; unifocal; unifocality; uni-centricity; and unicentric.
