# Supplementary material for: Comparing the outcome between multicentric/multifocal breast cancer and unifocal breast cancer: A systematic review and meta-analysis
Source: Front Oncol. 2022 Dec 16;12:1042789. doi: 10.3389/fonc.2022.1042789 (PMC9801517; doi:10.3389/fonc.2022.1042789)
Supplement: Supplementary file 2 [file Table_2.doc]

**Appendix Table 2** Details and characteristics of the included studies.

| First Author (year) | region | study design | Recruitment  period | sample size | Age  (year) | Median  follow-up  (months) | Method for determining MF/MC | Endpoint(s) reported | AJCC edition used for staging | MF/MC  definition |
| --- | --- | --- | --- | --- | --- | --- | --- | --- | --- | --- |
| Boyages (2010) | Australia | Retrospective | 1995-1995 | 848 | UFBC:  58.1  MMBC:  54.8 | 125 | Pathologic | BCSS | 5th | more than one pathologic invasive tumor described, irrespective of its location in the breast |
| Boros（2015） | Romania | Retrospective | 2002-2006 | 460 | NR | 104 (74-134) | Pathologic | OS | 7th | at least two histologically confirm- ed distinct invasive foci located within the same breast, detected at the same time and separated by “uninvolved” breast tissue, regardless of the distance between foci or localization  (in the same quadrant/in different quadrants) |
| Cabioglu (2009) | Turkey | Retrospective | 1990-2002 | 1322 | UFBC:  50 (21–97)  MMBC:  48 (27–97) | 55 | Pathologic | OS, DFS | 6th | the presence of simultaneous multiple macroscopically separated tumors in the same breast（MF:5mm＜d≤5cm） |
| Djordjevic-Jovanovic (2017) | Serbia | Retrospective | 2005-2007 | 584 | (mean ± SD)  MMBC:  54.7 ±11.9  UFBC:  53.9 ± 13.1 | NR | Clinical/ima-ging/patholo-gic | LRFS OS | NR | two or more separate tumors in the same quadrant or more than one quadrant of the same breast. |
| Duraker（2014） | Turkey | Retrospective | 1993-2002 | 3616 | UFBC:  49.0(20–86)  MMBC:  46.5 (26–77) | NR | Pathologic | DFS, LRFS, DMFS | 7th | presence of at least two macroscopically measurable invasive carcinomas separated by at least 5 mm of normal breast tissue in the same breast through random sampling during pathologic evaluation. |
| Egan (1982) | U.S. | Retrospective | 1965-1969 | 118 | NR | NR | Pathologic | OS | NR | wide separation grossly with noncancerous breast tissue intervening radiographically and microscopically; patterns of multiple areas spinkled throughout much of the breast; sharp delineation of different histologic types |
| El-Sheredy（2016） | Egypt | Retrospective | 2009-2009 | 140 | (mean ± SD)  MMBC:  46.95 ± 8.64  UFBC:  54.33 ± 8.76 | 50  (5-68) | Pathologic | DFS, OS | 6th | multiple, clearly separated, and macroscopically measurable tumor nodules in the same breast |
| Fowble (1993) | U.S. | Retrospective | 1982-1989 | 1352 | 54 (29–89) | 51 | Clinical/imaging/pathologic | OS, DFS | 2th | the clinical or mammographic presentation of two or more discrete tumors in the same breast |
| Fushimi (2019) | Japan | Retrospective | 2004-2006 | 734 | NR | 82 | pathologic | DFS, OS, DDFS | NR | MC/MF: ≥ 2 separate invasive unilateral breast tumors, irrespec- tive of any ductal carcinoma in situ (DCIS) between lesions,10 mm or more between them. |
| Joergensen (2008) | Denmark | Retrospective | 1996-2001 | 7024 | NR | 86 | Pathologic | OS, DFS | NR | more than one focus of invasive carcinoma separated by benign tissue whether in the same quadrant or in another. |
| Karakas (2018) | Turkey | Retrospective | 2003-2014 | 3890 | (mean)  MMBC:46.8  UFBC:49.7 | 56 | Pathologic | OS，DFS | 7th | multiple invasive lesions were found in the same quadrant of the breast or in more than 1 quadrant of the same breast |
| Katz（2001） | U.S. | Retrospective | 1975-1994 | 995 | (median- interquartile range)  48 (42–56) | 116 | Pathologic | LRR | NR | MF: two or more separate areas of invasive carcinoma within the same quadrant and/or separated by less than 4 cm;  MC: two or more areas of invasive disease in more than one quadrant of the breast and separated by at least 4 cm. |
| Kuan（2017） | Australia | Retrospective | 2000-2007 | 152 | (mean)  UFBC:57.2  MMBC:58.3 | UFBC:85.2  MMBC:78 | Pathologic | DRR, MR | 6th | MC/MF: the presence of two or more foci of invasive breast cancers, separated by either normal breast tissue or in situ disease identified either within one quadrant or within multiple quadrants of the breast respectively. |
| Kurtz（1990） | Switzerla-nd | Retrospective | 1975-1983 | 586 | NR | 71 | Clinical/imaging/patholo-gic | LRR, OS,  CBC | 2th | more than one discrete tumor mass /two or more tumor nodules separated by apparently non- neoplastic tissue. Tumors in which multifocality was apparent only on microscopic examination were classified as unifocal cancers. |
| Litton (2007) | U.S. | Retrospective | 1990-2002 | 300 | 32 (17–35) | 44 | Pathologic | OS, DFS | 5th | the presence of 2 or more foci of the same tumor clearly separated by normal tissue in the same breast |
| Lynch（2012） | U.S. | Retrospective | 1997-2010 | 3924 | NR | 51  ( 1–162 ) | Pathologic | BCSS, RFS, OS | 7th | MF and MC were defined as more than one lesion in the same quadrant or in separate quadrants, respectively. |
| Middleton（2002） | U.S. | Retrospective | 1983-1988 | 256 | 45 (28–69) | 72 | Pathologic | DFS | 5th | more than one separate focus of tumor in addition to the clinically identified (index tumor) located in a separate quadrant from the index tumor or centrally. |
| Neri（2015） | Italy | Prospective | 1991-2005 | 1158 | 63  (25–94) | 88  (11–248) | Pathologic | BCSS | NR | MF)：if there was more than one focus of invasive breast cancer separated by benign tissue in the same quadrant,  MC：when distinct tumor foci were found in different quadrants of the breast. |
| Mastropasqua（2020） | Italy | Retrospective | 2001-2006 | 170 | NR | pT1mi：108  pT1a：123 | Pathologic | LRR DMR | 8th | the presence of two or more invasive tumor foci separated from each other by normal breast tissue |
| Milulescu（2017） | Romania | Retrospective | 2007-2012 | 450 | UFBC:54  (24-81)  MFBC:44(36-79)  MCBC:56(35-72) | 36 | Pathologic | EFS | NR | lesions localized in the same quadrant/in different quadrants. Patients that presented with both MC and MF breast cancer, were defined as being multicentric. If a patient had both MF and MC diseases, they were classified as MC. |
| Ozturk（2021） | Turkey | Prospective | 2004-2017 | 1865 | NR | 58.5 | Pathologic | LRFS OS | NR | MF: having more than one tumor focus in the same quadrant,  MC: tumors refer to the presence of more than one tumor focus in different quadrants. |
| Pedersen（2004） | Denmark | Retrospective | 1985-1990 | 929 | (mean)  57.6 (27-94) | 126 | Pathologic | OS | NR | More than one focus of invasive carcinoma separated by benign breast tissue |
| Shaikh（2015） | U.S. | Prospective | NR | 373 | UFBC:  55（28–89）  MMBC:  54 (24–89) | 53 | Pathologic | DFS,OS，BCSS | 7th | MF：defined as multiple areas of disease localized within the same quadrant.；MC: multiple areas of disease within different quadrants or if there was a greater than 5 cm separation between areas of disease |
| Tan（2016） | Singapore | Retrospective | 2009-2011 | 160 | 48 (28–78) | 55 | Clinical/imaging/pathologic | LR | NR | MF: the presence of more than one focus of tumor within the same segment of the breast, MC: separate disease foci in different segments of the breast. |
| Tot（2011） | Sweden | Prospective | 1996-1998 | 574 | NR | 116 | Pathologic | BCSS, MR | 7th | the presence of multiple, well-delineated invasive tumor foci separated from each other by uninvolved breast tissue containing normal tissue, benign lesions, or in situ carcinoma, regardless of the distance between the foci. |
| Pekar（2013） | Sweden | Retrospective | 1996-1998 | 401 | NR | NR | Pathologic | BCSS | 7th | more than one well demarcated, invasive tumor focus was detected, with such foci separated from each other by normal breast tissue, benign lesions, or in situ carcinoma, regardless of the distance between foci. |
| Ustaalioglu（2012） | Turkey | Retrospective | 1994-2009 | 697 | 49  (22-82) | 26 | Pathologic | DFS, OS | 6th | MF: more than 1invasive tumors located in the same quadrant of the breast.  MC: 2 or more separate invasive tumors in different quadrant of the breast with a distance of at least 5 cm. |
| Wilson (1993) | U.S. | Retrospective | before 1988 | 1060 | NR | 71 | Clinical/imaging/patholo-gic | OS | NR | Having two or more separate lesions within the breast as judged by physical examination, mammography, visualization at the time of surgery, or grossly upon pathologic review. Patients found to have multifocal disease by microscopy only were not included, and normal-appearing breast tissue was grossly visualized between all lesions. |
| Yerushalmi（2009） | British | Prospective | 1989-2005 | 25320 | UFBC:  59 (49–69)  MMBC:  56 (47–67) | 82  (40-125) | Pathologic | CBC | NR | MF/MC: two or more invasive lesions anywhere in the same breast |
| Yerushalmi（2012） | British | Prospective | 1989-2005 | 19754 | NR | 95 | Pathologic | BCSS, OS | NR | two or more invasive lesions anywhere in the same breast based on pathology report. |
| Wolters（2013） | Germany | Retrospective | 1992-2008 | 8,935 | NR | NR | Pathologic | OS, RFS | 7th | being localized within the same quadrant/different quadrants of the breast. If patients showed both multicentric and multifocal breast cancer lesions, they were defined as multicentric. |

*Abbreviations: MMBC: multifocal and multicentric breast cancer; UFBC: unifocal breast cancer; OS: Overall Survival; DFS: Disease-free Survival; RFS: Relapse-free Survival; BCSS: Breast Cancer Specific Survival; LRFS: Locoregional Relapse-free Survival; LRR: local recurrences rate; DMR: distant metastases rate; DMFS: distant metastases free Survival; DDFS: distant disease-free survival; EFS: event free survival; D: distence between foic; MR: Mortality rates; DRR: Disease recurrence rates
