# Supplementary figures and images for: Comparing the outcome between multicentric/multifocal breast cancer and unifocal breast cancer: A systematic review and meta-analysis
Source: Front Oncol. 2022 Dec 16;12:1042789. doi: 10.3389/fonc.2022.1042789 (PMC9801517; doi:10.3389/fonc.2022.1042789)

**Appendix Figure 1 Risk of bias**

a. Risk of bias graph


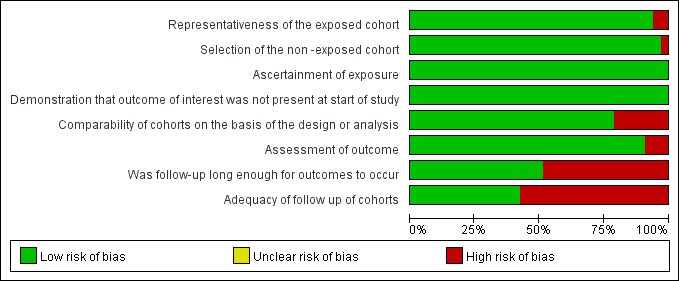


b. Risk of bias summary


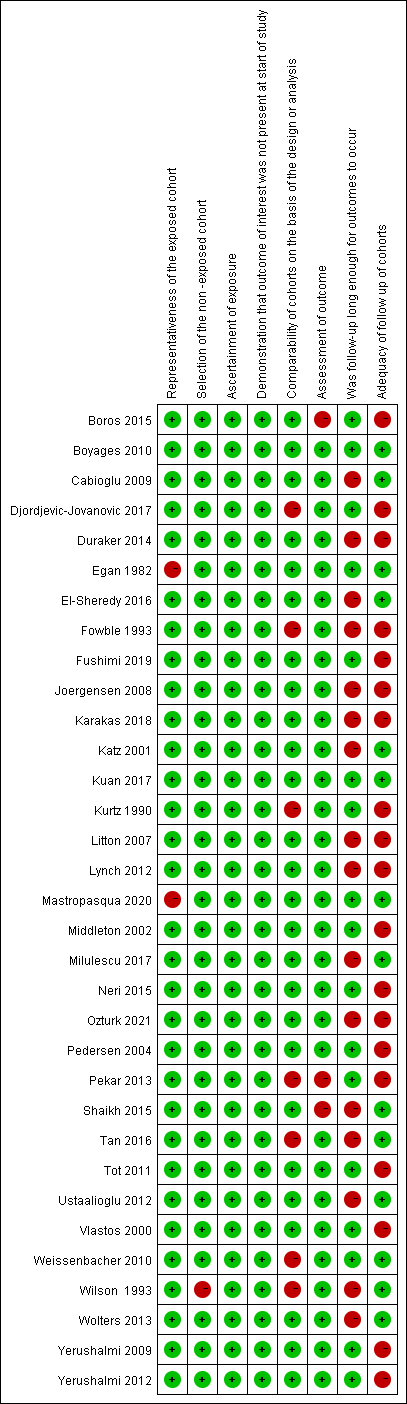

Supplement: Supplementary file 3 [file DataSheet_1.doc]
