# Supplementary material for: Comparing the outcome between multicentric/multifocal breast cancer and unifocal breast cancer: A systematic review and meta-analysis
Source: Front Oncol. 2022 Dec 16;12:1042789. doi: 10.3389/fonc.2022.1042789 (PMC9801517; doi:10.3389/fonc.2022.1042789)

**Appendix Figure 2** **Subgroup analysis on DFS and BCSS**

a. Subgroup analysis on DFS based on MMBC definition


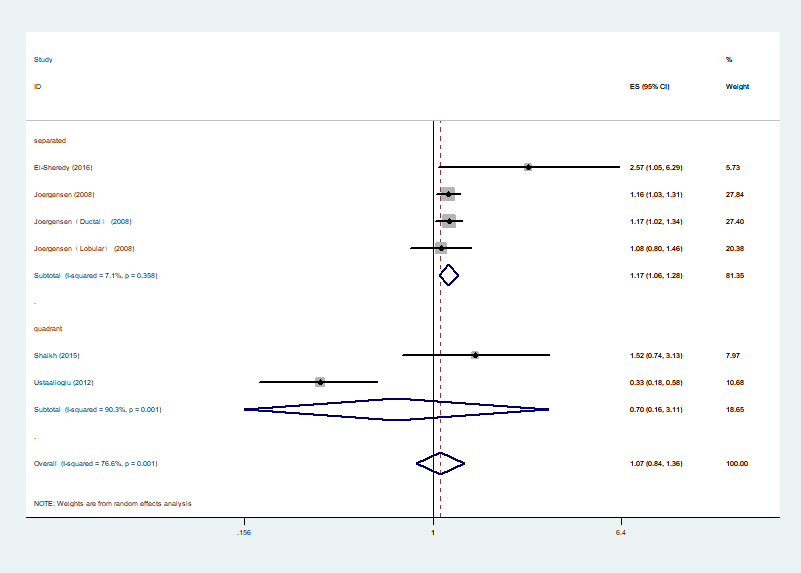


b. Subgroup analysis on DFS based on follow-up


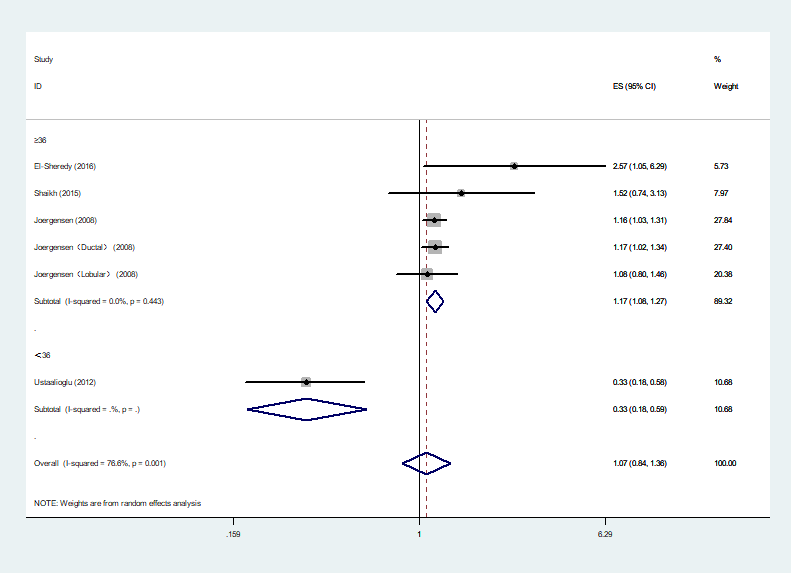


c. Subgroup analysis on DFS based on sample size


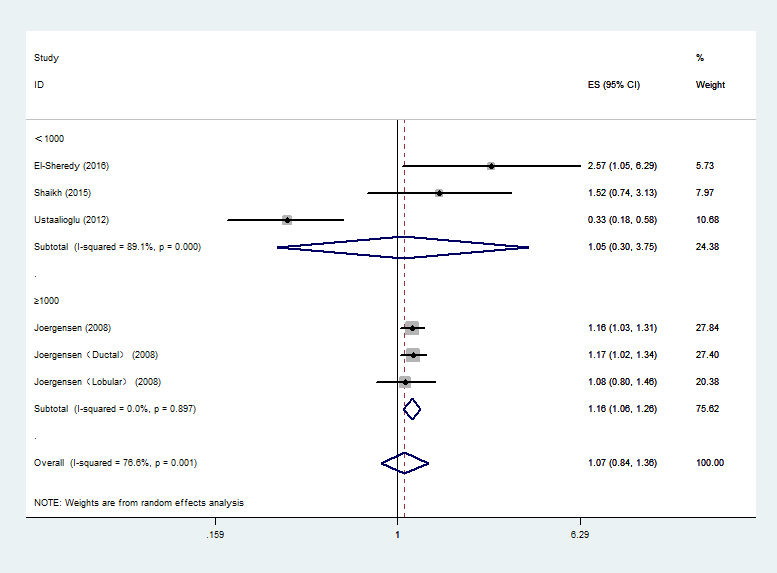


d. Subgroup analysis on BCSS based on sample size


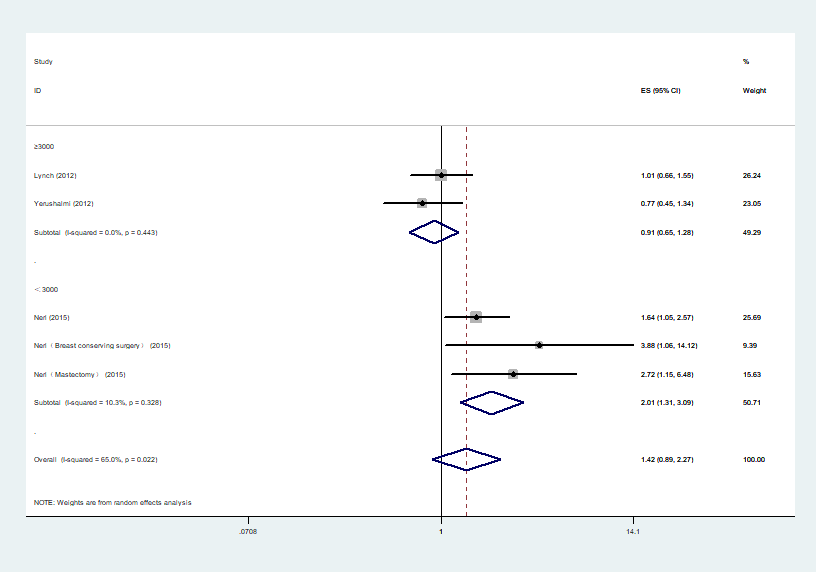


e. Subgroup analysis on BCSS based on MMBC definition


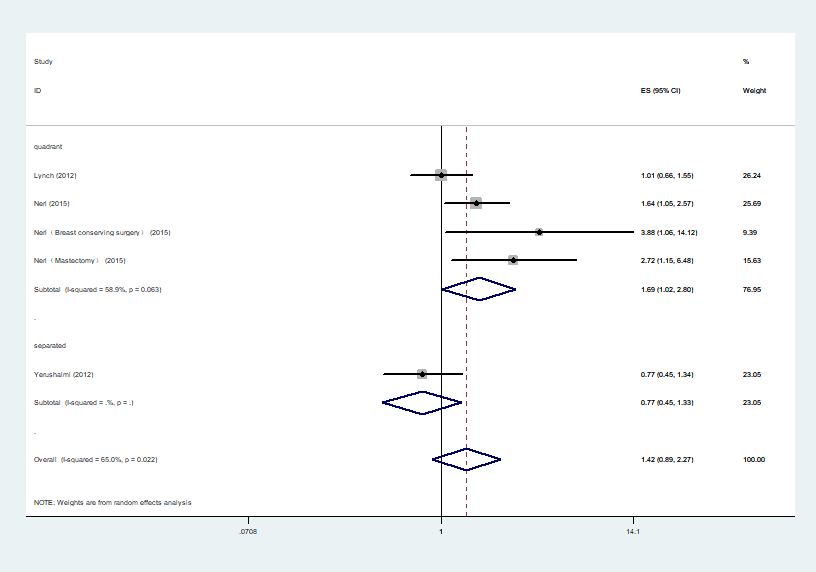

Supplement: Supplementary file 4 [file DataSheet_2.doc]
